# At-Home Evaluation of Both Wearable and Touchless Digital Health Technologies for Measuring Nocturnal Scratching in Atopic Dermatitis: Analytical Validation Study

**DOI:** 10.2196/72216

**Published:** 2025-07-15

**Authors:** Stefan Avey, Mark Morris, Davit Sargsyan, Molly V Lucas, Andrea O'Brisky, Kenneth Mosca, Andrew Elias, Nicholas Fountoulakis, Mehdi Boukhechba, Xuen Hoong Kok, Saiyam Jain, Mehrnoosh Oghbaie, Nikolay V Manyakov, Miao Wang, Zuleima Aguilar, Lynn Yieh

**Affiliations:** 1 Johnson & Johnson, 1400 McKean Rd, Spring House, PA, 19002, United States, 1 2157937355; 2 Johnson & Johnson, Raritan, NJ, United States; 3 Johnson & Johnson, London, United Kingdom; 4 Johnson & Johnson, Delhi, India; 5 Johnson & Johnson, Beerse, Belgium; 6 Johnson & Johnson, San Diego, CA, United States

**Keywords:** nocturnal scratch, scratching, itch, atopic dermatitis, wearable, touchless sensor, sensor, digital health technology, analytical validation, accelerometer

## Abstract

**Background:**

The most common symptom of atopic dermatitis (AD) is pruritus, which is often exacerbated at night and leads to nocturnal scratching and sleep disturbance. The quantification of nocturnal scratching provides an objective measure, which could be used as a clinical trial endpoint tracking this AD-related behavior. However, it is not clear how digital health technologies (DHTs) intended to measure scratching perform in the real-world environment of patient homes.

**Objective:**

In this study, we present the analytical validation of 2 DHTs: the GENEActiv wristband with Philips sleep and scratch algorithms (“Philips”) and the Emerald radio frequency touchless sensor (“Emerald”) to measure nocturnal scratching in adults with AD.

**Methods:**

Thirty-one participants (15 with moderate AD, 11 with mild AD, and 5 healthy volunteers) were enrolled in the study. Nocturnal scratching was assessed by each DHT in the study participant’s home environment over a 4-week observation period. Infrared videos were recorded during sleep twice per week and manually annotated for the intended sleep window (total sleep opportunity [TSO]) and scratching events. Human annotations for sleep and scratch measures were used as a reference for comparison with DHTs (“Reference”). Estimated TSO was compared for each DHT to Reference using Bland-Altman analysis. Within-night agreement of DHT-predicted scratching events versus the Reference was assessed by sensitivity, precision, and *F*_1_-score. Intraclass correlation was used to compare night-level scratch summaries (scratch duration per hour of TSO and scratch frequency per hour of TSO) between each DHT and the Reference.

**Results:**

Characterization of human-annotated scratching revealed a basal level of scratching in both healthy volunteers (13.1 seconds per hour) and in participants with AD on nights when no itch was reported (10.2 seconds per hour). The TSO window was quantified accurately with both DHTs having a mean bias compared with Reference of <30 minutes. The within-night agreement with reference to scratch detection performance resulted in *F*_1_-scores at the disease group level ranging from 0.51 to 0.68 for the Emerald DHT and 0.47 to 0.56 for the Philips DHT. The night-level agreement of nocturnal scratch duration and frequency with human raters fell mostly in the moderate—good range of intraclass correlation coefficients (0.5‐0.9) in participants with AD and was not significantly lower than the level of agreement between any 2 human raters.

**Conclusions:**

These results support the analytical validity of both DHTs tested for continuous measurement of nocturnal scratching in individuals with AD in the home environment. Opportunities remain for improving the performance of the DHTs tested, especially in the precision of wrist-worn accelerometer scratch detection, to reach human-level performance. Additional data collection in diverse patient populations will be beneficial for practitioners intending to use or improve these tools for quantifying nocturnal scratching behavior.

## Introduction

Atopic dermatitis (AD) is an inflammatory skin disease that has been reported to affect up to 10% of the adult population [1]. The most common symptom is pruritus or itch. Itch is reported to be exacerbated at night [2], and the consequent scratching often disrupts sleep and can be accompanied by anxiety, depression, and even suicidal ideation [3]. While there are patient-reported outcomes (PROs) to assess pruritus (the sensation of itch), these are not designed to measure the amount of scratching behavior [4,5]. Nocturnal scratching, which by definition is an action of rhythmic and repetitive skin contact movement performed during a delimited time period of intended and actual sleep [6], is unlikely to be accurately self-reported and has been shown to correlate only weakly with self-reported measures of itch [7]. Therefore, an objective measurement of nocturnal scratching would complement a patient-reported measurement of itch in future clinical trials.

Video recording followed by manual human annotation of scratch events is considered the reference standard for assessment of nocturnal scratching [7] and has been used as a research tool for more than 25 years to characterize scratching in AD [8,9]. However, the privacy implications of collecting video as well as the time-intensive manual annotation process preclude the widespread assessment of nocturnal scratching in clinical trials. Recently, several digital health technologies (DHTs) have been developed that claim to objectively quantify nocturnal scratching, which could help facilitate adoption of nocturnal scratching as an objective and readily measured clinical trial end point [7,10-18]. The most widely studied type of technology for measuring scratching is actigraphy (wrist-worn accelerometers). Based on previous research, it is well established that actigraphy-based methods have poor sensitivity for detecting finger-dominant scratching or other types of non–wrist-driven scratching, such as scratching one leg against the other [12]. Recently, touchless sensors have been proposed as a tool for capturing a wider variety of scratching movements without requiring any physical contact with the body. Indeed, touchless sensors using reflections of radio waves have already shown promise in assessing human behaviors such as sleep [19,20] and gait [21].

This study builds on existing research in the field that has developed algorithms for detecting nocturnal scratching from either wrist-worn accelerometers or touchless sensors in a clinical setting by showing how they both perform in comparison with video observation in individuals’ natural sleeping environment. To date, there have been only a few studies that examined the analytical validity of DHT-derived nocturnal scratch measures in a home setting, and these have concentrated on pediatric patients with AD [22,23]. In contrast, this study provides independent analytical validation evidence for DHT-derived nocturnal scratch measures in the home environment of adults with AD, using both a wrist-worn wearable and a touchless sensor.

This analytical validation study was designed to evaluate two DHTs: (1) the GENEActiv wristband with Philips sleep and scratch algorithms (referred to hereafter as “Philips”) and (2) the Emerald radio frequency (RF)-based sensor with Emerald sleep and scratch algorithms (referred to hereafter as “Emerald”). The performance of the 2 DHTs to measure nocturnal scratching during the total sleep opportunity (TSO) window was independently compared against human-labeled infrared (IR) camera recording data (referred to hereafter as “Reference”). The comparison to Reference was done both within nights and on nightly level summaries: normalized scratch frequency (number of scratch bouts per hour of TSO) and normalized scratch duration (seconds of scratch per hour of TSO). We begin by describing the study and patient population, then we characterize the scratch behavior in the patient cohorts, followed by a comparison of the TSO window estimates, then move to comparing scratch classification performance within nights, and finally to comparing the nightly scratch measures between each DHT and Reference.

## Methods

### Study Design

**Figure 1.**
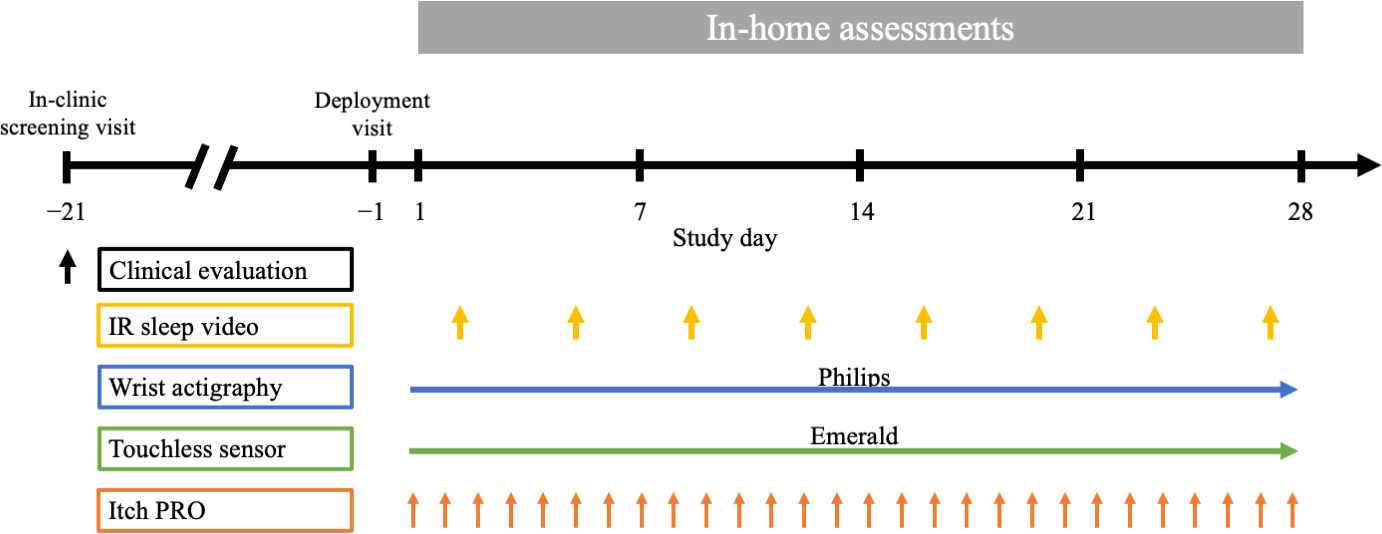
Study design. Clinician-reported (vIGA-ADTM, EASI, and SCORAD) and 1 patient-reported outcome (peak nocturnal pruritus numeric rating scale) were assessed at the in-person screening visit. The deployment visit on study day 1 consisted of the installation of the touchless sensor, IR camera, and assignment of the wristbands for wrist actigraphy. Nocturnal scratch measures were assessed nightly during the observation period with both the Philips and Emerald DHTs. IR sleep videos were recorded on any 2 nights per week, and video observers labeled sleep and scratch measures. Itch was assessed daily with the Atopic Dermatitis Itch Scale. IR: infrared; PRO: patient-reported outcome.

The study included an in-clinic screening visit, a screening period of up to 3 weeks, and a 4-week observation period at each participant’s home. Clinical scores for characterizing the severity of disease in patients with AD were assessed during the in-clinic screening visit. The in-home portion of the study included a 28-day observation period during which scratching was assessed every night by the Philips and Emerald DHTs (Figure 1).

### Study Population

The study enrolled men and women, aged 18 years and older, with moderate to severe AD, mild AD, or healthy volunteers (HVs). Participants with AD were required to have an established diagnosis for at least 12 months and meet the following key inclusion criteria at screening: patients were eligible for the mild AD cohort if they had a Validated Investigator Global Assessment scale for Atopic Dermatitis (vIGA-AD) score of 2 and ≥3% body surface area of AD involvement; and patients were eligible for the moderate to severe AD cohort if they had a vIGA-AD score of ≥3, ≥10% body surface area of AD involvement, an Eczema Area and Severity Index score of ≥16, and an excoriation component score of >0 in at least 1 body region. While patients with moderate to severe AD based on vIGA-AD score were eligible, those enrolled in this cohort all had AD of moderate severity according to vIGA-AD (vIGA-AD score of 3). Therefore, this group of participants is referred to as a “Moderate AD cohort.” Participants with AD were allowed to continue treatment for AD as long as they were on a stable regimen by the start of the observation period, and topical medications were permitted throughout the study for the treatment of AD flares. HVs were eligible if they were healthy on the basis of medical history at screening, did not have chronic itch, and reported a numeric rating scale (NRS) Peak Nocturnal Itch score of ≤1 (over a 7-day recall period) at screening.

### Reference Data Generation

Video recordings during sleep were captured in each participant’s home environment using a bespoke IR video camera developed by Emerald Innovations Inc. The camera consisted of an IMX219 8-megapixel sensor and an IR illuminator setup capable of capturing images in 720-pixel resolution at 21 frames per second. The camera was mounted on the wall at the head of the bed about 1 m above the bed surface. Participants were asked to record video during sleep hours for 8 nights over the 4-week observation period (ie, 2 nights per week).

Videos were masked by blurring faces before subsequent processing and labeling. A computer vision–based motion-filtering algorithm trained on historical data was applied to the masked video before human labeling. This algorithm automatically removed video segments containing no motion to reduce the duration of video presented for labeling to the human raters. Random spot checks were performed on video data from each participant to confirm that the motion-filtering algorithm did not inadvertently filter out any participant motion.

Independent raters watched the videos and provided labels for multiple tasks.

1. **Scratching bout labeling:** Two independent raters (R1 and R2) watched a video segment and labeled the start and end times of all scratching behavior episodes (defined as “any repetitive and rhythmic skin contact movement—including through fabric—performed with any part of the body”). Consensus was reached in the first round if the intersection over union of R1 and R2 annotations exceeded a threshold of 0.765 (empirically chosen based on historical rating data). If consensus was not reached, the video segment was rated by 2 additional raters (R3 and R4) in round 2 and the same consensus threshold was used. If consensus was reached in either round 1 or 2, the intersection of the 2 raters’ labels within the round reaching consensus was taken as the final Reference. If consensus was still not reached after round 2, a fifth rater (R5) decided on the final scratch labels in round 3. Raters had access to view labels from all previous rounds, but labels were independent within a given round.

2. **TSO labeling**: Two independent raters (R1 and R2) watched the full night video and determined when the participant got into bed intending to sleep (TSO start) and when the participant got out of bed for the last time, intending to stay awake (TSO end). If the 2 raters in round 1 did not agree (disagreement defined as >1-minute difference in either TSO start or TSO end), a third rater with access to labels from round 1 determined the TSO start and end labels.

### Emerald Data Collection and Scratch Algorithm

The Emerald system consists of a sensor using frequency-modulated continuous wave radar and antennae arrays to receive reflections from nearby people. The Emerald RF system uses a machine learning algorithm that operates on the data recorded by the Emerald sensor to derive sleep-related metrics [19,20]. It uses a second proprietary machine learning algorithm that operates on the recorded data to derive scratching-related metrics [24]. In this study, the RF sensor was installed on the bedroom wall 1.15 m from the floor and not more than 5 m from the participant’s bed. Participants in this study agreed to be the only person sleeping in the room with the RF sensor and agreed to keep pets out of the bedroom during the 28-day observation period.

### Philips Data Collection and Scratch Algorithm

The GENEActiv Original (ActivInsights) wristband was configured to collect accelerometer data at 20 Hz. Each participant received 2 wristbands on study day 1 and wore both for 14 days. Each participant received a new pair of wristbands around day 14 and shipped the first set back to study sites for data collection. Participants were instructed to wear 1 wristband on the nondominant wrist continuously and the other on the dominant wrist during sleep hours.

Philips Respironics Rapid Actigraphy Data Analyzer (RADA) algorithms were applied to the raw GENEActiv data to generate sleep and scratch metrics. The RADA scratch algorithm is based on a previously published study [12]. Scratches output by the RADA algorithm were characterized as left wrist, right wrist, or both wrists. The analysis of the data was wrist agnostic, and all scratching events were used for analysis.

### Outcome Definitions

TSO was defined for the Reference by the human-labeled TSO start and TSO end times from video. TSO was calculated separately for each DHT to emulate how the DHT would be used in a future study (ie, without information from the videos). TSO for the Philips DHT was selected as the major rest period on each night as determined by the RADA algorithms. TSO for the Emerald DHT was calculated as the merged sleep period where multiple sleep periods on the same night with <30 minutes gap were merged. Any TSO with a duration of <3.5 hours was excluded from the analysis as they were not considered representative of a nightly sleep period. A short, estimated TSO can occur when the true TSO is short, or the estimated TSO is capturing only a portion of the true TSO. The cutoff was set based on the observed data to minimize filtering out any true TSO. The intent of the filter was to automatically detect and remove cases when only a portion of the true TSO was detected (eg, the video was switched on only for a portion of the true TSO). Seven out of 232 Reference nights (3%) were excluded from all analyses due to Reference TSO of <3.5 hours. One additional night was excluded when comparing Reference to Philips, and 3 additional nights were excluded when comparing Reference to Emerald due to the respective DHT-derived TSO of <3.5 hours. These 11 nights impacted by the TSO filter on nights with Reference data were examined, and in 8 cases, the short TSO is a subset of a longer TSO detected by another method; in 2 cases, the short TSO does not overlap at all with the other methods, and in 1 case, all methods of TSO determination were short. Over all nights recorded by the DHTs, 23 out of 728 nights (3%) were removed for short TSO from Emerald, and 11 out of 635 (2%) nights were removed for short TSO from Philips.

The 2 primary scratch outcomes analyzed were the scratch duration and the scratch frequency. Before any downstream analysis, individual scratch events were merged and filtered. First, all scratch events with a <3-second gap were merged into a single scratching bout. Next, only scratch events of at least 2 seconds in duration (69% of all Reference scratch events) were retained. The same merging and filtering of scratch events were applied to both DHTs and the Reference. A minimum duration of 2 seconds was chosen both because the clinical relevance of very short scratch events is unclear and to allow for fair comparisons since 2 seconds was the minimum duration of scratch predicted by the Philips algorithm [12]. In all analyses, scratch duration and frequency were normalized by the duration of the TSO window so that changes in sleep time would not unduly impact the outcomes. The scratch duration is presented in units of seconds of scratch per hour of TSO, while the scratch frequency is presented in units of number of scratch bouts per hour of TSO. Both scratch duration and scratch frequency have right-skewed distributions and are therefore modeled (in intraclass correlation coefficient [ICC] analysis) on a logarithmic scale.

### Patient-Reported Outcomes

Atopic Dermatitis Itch Scale was completed in the AD cohort twice daily (morning and evening) to assess both nighttime and daytime itch. The characterization of scratch by itch level was performed using the morning Atopic Dermatitis Itch Scale administration, which asked about worst eczema-related itching last night on a 0‐10 numeric rating scale. PROs were administered electronically through a mobile phone app. In cases of technical issues with the phones, paper PROs were completed, and responses were entered by the site staff. Only responses with time stamps (electronic or handwritten) between 4 AM and 12 PM were considered valid for the morning administration. In rare cases where multiple responses were recorded on the same day, all responses on that day were excluded from analysis.

### Statistical Analysis

#### Characterization of Nocturnal Scratching Behavior

Descriptive statistics were used to characterize the scratching behavior in various subgroups of participants (or participant nights). All scratching characterization was done using human-labeled scratching. To summarize scratching behavior at the participant level, first, the night-level metrics were calculated for all nights with labeled scratches. Then, the geometric means were calculated at the participant level for each scratch metric over all nights. Box plots were used to summarize the distribution and medians of the distributions were compared between subgroups. Data were visualized on a linear scale in original units for ease of interpretation.

#### Bland-Altman Analysis of TSO

Bland-Altman analysis was performed accounting for repeated measures using a mixed-effects model. Observations without paired data due to missing data in either Reference or DHT were removed from the analysis, and the number of paired observations (participant nights) is shown in the figures.

#### Within-Night Comparison to Reference

The time period considered for within-night comparison to Reference was the intersection of the TSO between the DHT and the Reference. Nonoverlapping 10-second windows (nonoverlapping 1-second windows for the sensitivity analysis) from all nights and all participants within a cohort were categorized as true positives, false positives, true negatives, or false negatives and combined at the cohort level to generate a confusion matrix and subsequent performance metrics. A window was considered positive for the event if any part of a scratch event overlapped the window. *F*_1_-score was defined as the harmonic mean of sensitivity and precision for the positive class (windows containing scratch).

When comparing *F*_1_-score performance by participant characteristics, the variables considered were age, BMI, biological sex, ethnicity (ignoring 2 cases when ethnicity was not reported), race (converted to 2 classes “White” and “non-White” due to sample size and ignoring 2 cases where race was not reported), scratch prevalence, total epochs, and power of scratch events (for Philips DHT only). The total epochs are the number of 10-second windows pooled across all nights for each participant. The scratch prevalence is the percentage of the total epochs containing Reference scratch events and is calculated as the sum of true positives and false negatives divided by the total epochs. The power of Philips-predicted scratch events was calculated as the geometric mean of the power (in G) for all events for a given participant before any merging or filtering of scratch events. A 2-sided *t* test was performed for categorical variables, and a Pearson correlation coefficient was estimated for continuous variables. Nominal *P* values were reported.

#### Intraclass Correlation Analysis

ICCs were used to compare night-level metrics between each DHT and human raters. ICCs were estimated using outputs from linear mixed-effects models (function *lmer* from R package *lmerTest* (version 3.1-3 [25]) on log-transformed scratch outcomes (normalized scratch frequency and normalized scratch duration) as the response variables, scratch metric source (Philips, Emerald, Reference) as the fixed effect, a random intercept for study participant, and a random intercept for study day nested within participant. Function *icc* (R package *performance* version 0.12.0 [26]) was used to calculate ICCs; 95% CIs for the ICC estimates were calculated by taking 500 bootstrap samples. This form of ICC calculation corresponds to “Case 3A: Two-way mixed effects model, interaction absent” with an absolute agreement definition using the McGraw and Wong convention [27].

### Ethical Considerations

This clinical study was performed at 6 commercial dermatology clinic sites across the United States between 2022 and 2023. Institutional review board approval was received for the study (WCG institutional review board protocol no 20213992), and all study participants provided informed consent. This study adhered to ethical standards by obtaining informed consent from all participants and ensuring that they received fair compensation for their involvement. Participant privacy and confidentiality were protected as outlined in the informed consent process, and any participant-level data presented in this manuscript are pseudonymized to preserve participant privacy.

## Results

### Study Design and Participant Characteristics

Thirty-one participants enrolled in the study, including 15 with moderate AD, 11 with mild AD, and 5 HVs. Of the 31 participants, 2 participants were excluded for not meeting the inclusion or exclusion criteria (one based on disease severity and another based on change in therapy during the screening period). One additional participant in the moderate AD group was excluded from the analysis due to capture of poor-quality videos that failed spot checks and could not be manually labeled. A schematic overview of the study design is shown in Figure 1.

A summary of demographics and disease severity characteristics at baseline for the 28 participants included in the analysis is shown in Table 1. Participants ranged in age from 19 to 65 (median 37.5) years. Participants were categorized as having mild AD or moderate AD based on the vIGA-AD. Mean Eczema Area and Severity Index scores for the moderate AD and mild AD groups were 17.54 and 4.63, respectively. Mean SCORing Atopic Dermatitis (SCORAD) values for the moderate AD and mild AD groups were approximately 50 and 36, respectively. Peak nocturnal itch reported on a 0‐10 NRS was 0 at screening for all HVs and at least 2 for all patients with AD, with a similar median peak nocturnal itch score for the groups with mild and moderate AD (mild AD: 6.0, moderate AD: 5.5).

**Table 1.** Summary of demographic and disease characteristics at screening.

Analysis set: per protocol set	HVs^a^ (n=5)	Moderate AD^b^ (n=14)	Mild AD (n=9)	Total (n=28)
Age, years				
Mean (SD)	27.20 (9.65)	42.21 (15.39)	43.89 (10.91)	40.07 (14.18)
Median (range)	25 (19-42)	38.5 (21-65)	47 (28-56)	37.5 (19-65)
					Sex, n (%)				
Female	3 (60.00)	9 (64.29)	5 (55.56)	17 (60.71)
Male	2 (40.00)	5 (35.71)	4 (44.44)	11 (39.29)
Race, n (%)				
American Indian/Alaskan Native	0 (0.00)	2 (14.29)	0 (0.00)	2 (7.14)
Asian	0 (0.00)	1 (7.14)	1 (11.11)	2 (7.14)
Black/ African American	0 (0.00)	1 (7.14)	2 (22.22)	3 (10.71)
White	5 (100.00)	9 (64.29)	4 (44.44)	18 (64.29)
Multiple	0 (0.00)	0 (0.00)	1 (11.11)	1 (3.57)
Not reported	0 (0.00)	1 (7.14)	1 (11.11)	2 (7.14)
Ethnicity, n (%)				
Hispanic or Latino	3 (60.00)	7 (50.00)	1 (11.11)	11 (39.29)
Non-Hispanic or Latino	2 (40.00)	6 (42.86)	7 (77.78)	15 (53.57)
Not reported	0 (0.00)	1 (7.14)	1 (11.11)	2 (7.14)
NRS^c^ peak nocturnal itch				
Median (range)	0.00 (0.00-0.00)	5.50 (2.00-8.00)	6.00 (2.00-9.00)	5.00 (0.00-9.00)
EASI^d^				
Mean (SD)	N/A^e^	17.54 (0.97)	4.63 (2.86)	12.49 (6.71)
Median (range)	N/A (N/A)	17.25 (16.40-20.00)	4.20 (0.60-11.00)	16.80 (0.60-20.00)
IQR^f^	N/A	16.92-17.98	3.20-5.00	4.65-17.40
SCORAD^g^				
Mean (SD)	N/A	50.27 (8.21)	35.87 (11.03)	44.63 (11.65)
Median (range)	N/A (N/A)	51.10 (36.50-66.90)	35.00 (18.20-52.60)	47.90 (18.20-66.90)
IQR	N/A	47.23-53.45	32.00-40.80	36.95-52.70
vIGA-AD^h^, n (%)				
Clear (0)	N/A	0 (0.00)	0 (0.00)	0 (0.00)
Almost clear (1)	N/A	0 (0.00)	0 (0.00)	0 (0.00)
Mild (2)	N/A	0 (0.00)	9 (100.00)	9 (39.13)
Moderate (3)	N/A	14 (100.00)	0 (0.00)	14 (60.87)
Severe (4)	N/A	0 (0.00)	0 (0.00)	0 (0.00)

a HVs: healthy volunteers.

b AD: atopic dermatitis.

c NRS: numeric rating scale.

d EASI: Eczema Area and Severity Index.

e N/A: not applicable.

f IQR: interquartile range.

g SCORAD: SCORing Atopic Dermatitis.

h vIGA-AD: Validated Investigator Global Assessment scale for Atopic Dermatitis.

### Characterization of Nocturnal Scratching Behavior

To define a nocturnal scratch outcome, it was first necessary to determine the time window during which scratching was to be measured. Following the terminology used in previous research, we call this window of intended sleep the TSO [10]. The TSO windows for the Reference were manually annotated and defined to start when the participant got into the bed intending to sleep and defined to end when the participant got out of the bed intending to stay awake.

**Figure 2.**
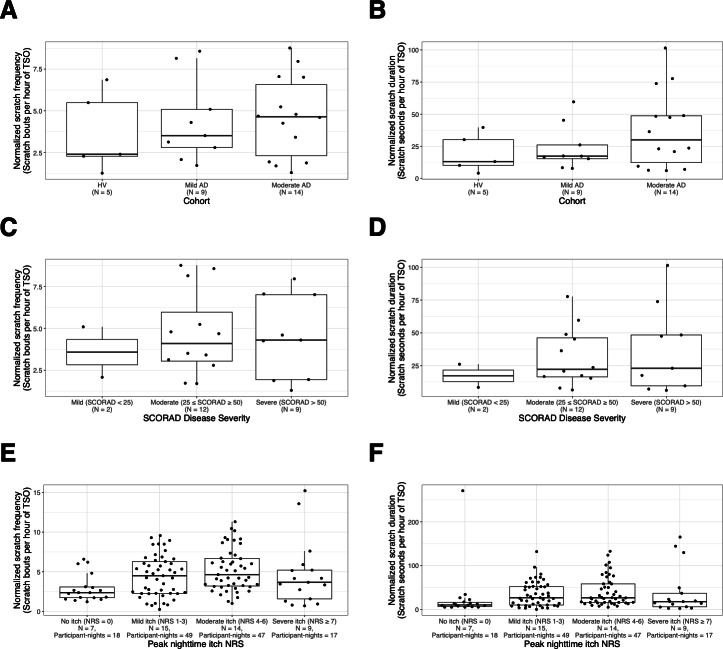
Characterization of human-labeled scratching behavior by disease severity and patient-reported itch. Distribution of normalized scratch frequency (scratch bouts per hour; left column) and normalized scratch duration (scratch seconds per hour; right column) are shown. Participant level average (**A**) scratch frequency and (**B**) scratch duration by cohort. Participant level average (**C**) scratch frequency and (**D**) scratch duration by SCORAD severity within the AD cohort only. Participant-night level (**E**) scratch frequency and (**F**) scratch duration by peak nighttime itch NRS obtained via the Atopic Dermatitis Itch Scale morning administration within the AD cohort only. Box plots show the IQR of the distribution. Bold horizontal lines within the box show the median. Whiskers show the maximum and minimum values after excluding any outliers. The box plot is overlaid with all observed data. AD: atopic dermatitis; ADIS: Atopic Dermatitis Itch Scale; HV: healthy volunteer; NRS: numeric rating scale; SCORAD: SCORing Atopic Dermatitis; TSO: total sleep opportunity.

Nightly scratch measures (normalized scratch duration and normalized scratch frequency) as defined in the Reference were characterized by disease severity and patient-reported itch in Figure 2. The median frequency and duration of nighttime scratching increase with AD disease severity from HVs (2.4 bouts per hour, 13.1 seconds per hour) to mild AD (3.51 bouts per hour, 17.4 seconds per hour) to moderate AD (4.64 bouts per hour, 30.0 seconds per hour); however, the ranges are highly overlapping between all 3 cohorts (Figures 2A and B). Scratching was also characterized by SCORAD severity since this is a frequently used scoring system in AD. Similarly to cohort, there is a trend of increasing median nocturnal scratch frequency and duration with increasing severity of SCORAD but highly overlapping ranges (Figures 2C and D).

Finally, we investigated how the reference nocturnal scratch frequency and duration compared on nights when participants reported no itch, mild itch, moderate itch, or severe itch (Figures 2E and F). In this cross-sectional analysis, when AD study participants reported in the morning that the previous night had no itch, the scratching was dramatically lower (but not zero) than when any itch was reported. Median scratch frequency on nights with no itch was 2.37 bouts per hour versus 4.5 bouts per hour with mild itch and 4.64 bouts per hour with moderate itch. The trend did not hold for severe itch, which had the lowest number of nights and fell in between the levels of no itch and mild itch with a median of 3.68 bouts per hour. Similar trends were observed for nocturnal scratch duration with 10.2 seconds per hour in no itch, 26.7 seconds per hour in mild itch, 26.5 seconds per hour in moderate itch, and 17.8 seconds per hour in severe itch.

### TSO Agreement With Reference

**Figure 3.**
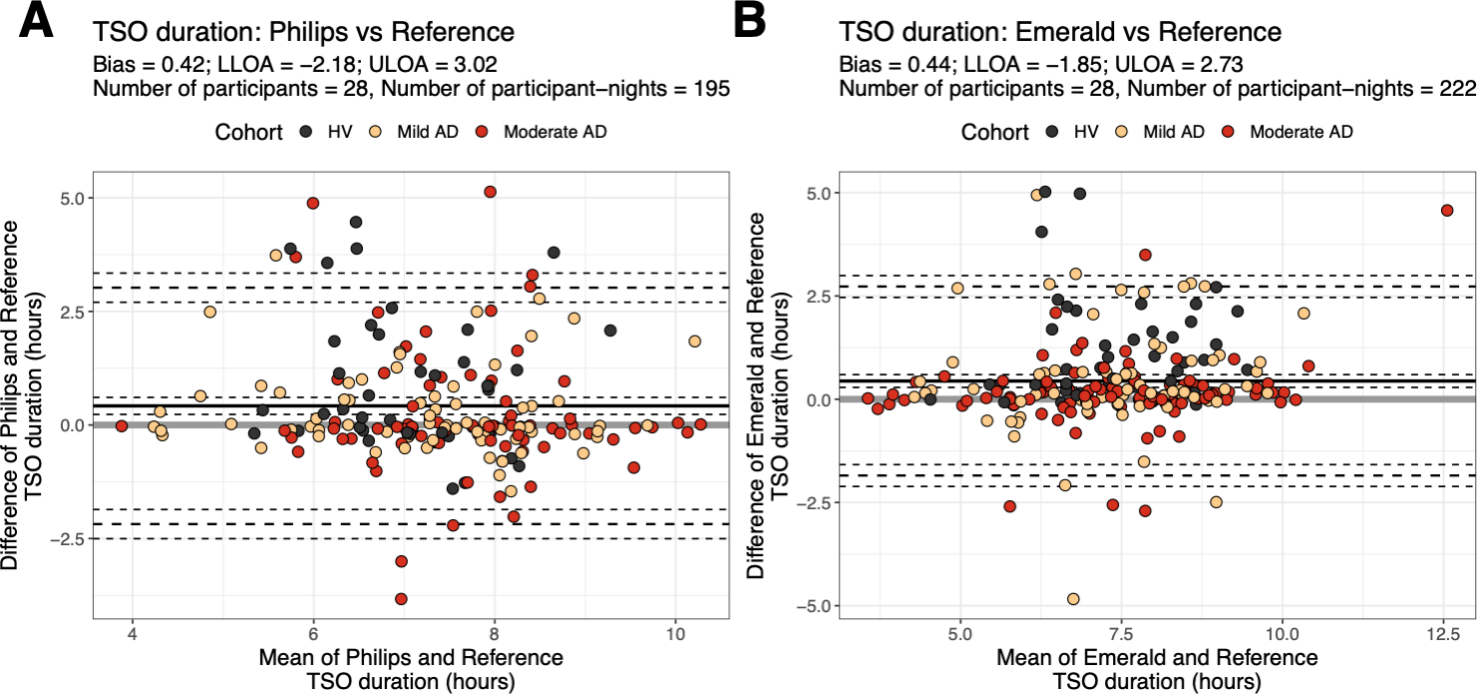
Bland-Altman plots of TSO duration. Bland-Altman plots for (**A**) Philips versus Reference and (**B**) Emerald versus Reference. Each point represents a single night from 1 participant. Cohorts are represented by different colors. The solid black horizontal line is the bias, and the long dashed horizontal lines are the 95% LLOA and ULOA. The short dashed horizontal lines are the 95% CI lower and upper bounds for the bias and the 95% limits of agreement. AD: atopic dermatitis; HV: healthy volunteer; LLOA: lower limit of agreement; TSO: total sleep opportunity; ULOA: upper limit of agreement.

After characterizing the scratching behavior with the human-labeled reference, we began to assess the agreement of the DHT-derived measures with the Reference. First, we assessed the level of agreement between TSO derived from Philips versus Reference and from Emerald versus Reference with a Bland-Altman analysis (Figures 3A and B). The average bias in TSO in the home environment was less than half an hour for both DHTs with bias (lower and upper 95% limits of agreement) estimates of 0.42 hours (−2.18 hours and 3.02 hours) for Philips and 0.44 hours (−1.85 hours and 2.73 hours) for Emerald.

### Within-Night Scratch Detection Performance

Next, we assessed the level of agreement in scratch classification between each DHT and the Reference for all 10-second windows. The time period considered for the evaluation was the intersection of the TSO between the Reference and the corresponding DHT to avoid ambiguous cases where scratch labels were missing. All time windows within the TSO intersections were considered, and no balancing of the scratch and nonscratch windows was performed. In the moderate AD group, an *F*_1_-score of 0.64 was achieved for Emerald and 0.43 for Philips (Table 2). Overall, the performance of the Emerald DHT, as measured by *F*_1_-score, was noticeably higher than that of the Philips DHT in each cohort. The difference in *F*_1_-scores was mainly due to the lower precision (0.29‐0.36) of the Philips algorithm, indicating that many false-positive scratching events were identified.

**Table 2.** Sensitivity, precision, *F*_1_-score, and balanced accuracy to detect scratching events on 10-second windows compared to the Reference.

	Emerald	Philips
HVs^a^		
N (participants)	5	5
N (participant nights)	46	45
Sensitivity	0.52	0.69
Precision	0.61	0.44
*F*_1_-score	0.56	0.54
Balanced accuracy	0.76	0.84
Mild AD^b^		
N (participants)	9	9
N (participant nights)	76	73
Sensitivity	0.54	0.62
Precision	0.49	0.38
*F*_1_-score	0.51	0.47
Balanced accuracy	0.76	0.80
Moderate AD		
N (participants)	14	14
N (participant nights)	98	74
Sensitivity	0.71	0.72
Precision	0.66	0.46
*F*_1_-score	0.68	0.56
Balanced accuracy	0.85	0.85

a HVs: healthy volunteers.

b AD: atopic dermatitis.

To better understand the behaviors leading to false-positive scratch prediction with the Philips algorithm, we sampled 2 nights from each study participant and watched the videos during the false-positive event to identify which nonscratch movements were contributing to false-positive scratch predictions. We found that the main nonscratch movements incorrectly classified as scratch were body repositioning within the bed, mobile phone usage, and movement associated with entering or exiting the bed.

A more detailed listing of performance metrics by participant is shown in Multimedia Appendix 1. A 10-second window size was chosen for this analysis of within-night performance to mitigate any misalignment of scratch events resulting from incomplete clock synchronization between DHTs and video. A sensitivity analysis showing the performance metrics with 1-second windows is shown in Multimedia Appendix 2. Overall, the trends observed with 10-second windows are consistent with 1-second windows, but the performance metrics are generally lower.

Because of the wide range of participant-level *F*_1_-scores observed (ranging from 0.13 to 0.85), we investigated whether any participant characteristics were associated with *F*_1_-scores (Multimedia Appendix 3). We found that the strongest factor associated with *F*_1_-score was the scratch prevalence that varied between 0.5% and 4.5% when pooling all epochs at the participant level. Scratch prevalence was strongly positively correlated with *F*_1_-score for both Philips (*r*=0.82; *P*<.001) and Emerald (*r*=0.59; *P*=.001) DHTs. Ethnicity was the next most associated variable with *F*_1_-score in this dataset, with non-Hispanic or non-Latino participants having an average *F*_1_-score 0.17 lower than Hispanic or Latino participants (Philips: *P*=.004, Emerald: *P*=.01). However, upon closer inspection of the variables’ correlation structure, it was noted that ethnicity is associated with prevalence in this dataset, which is likely driving the observed association. We also identified a significant correlation between the participants’ geometric mean power of scratch events (in G) and the *F*_1_-score for the Philips DHT (*r*=0.43; *P*=.02). Additionally, a negative correlation was observed between participant age and *F*_1_-score (Philips: *r*=−0.39, *P*=.04; Emerald: *r*=−0.41, *P*=.03). Participant age was found to be negatively correlated with the power of scratching events, which may be partly mediating the association with age.

### Agreement of Nightly Scratch Measures With Reference

**Figure 4.**
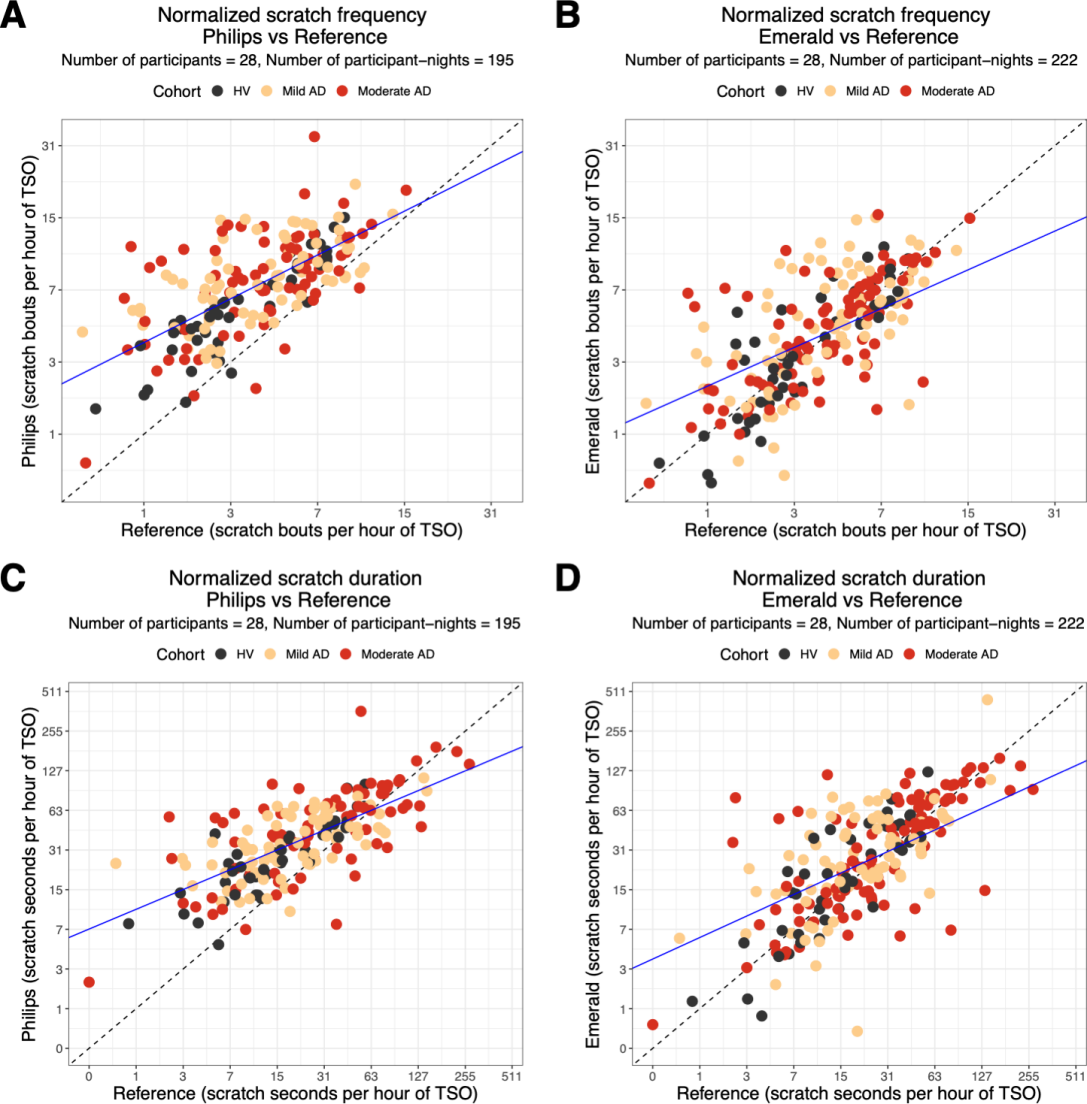
Scatter plots of night-level scratch metrics in each DHT versus Reference. Normalized scratch frequency of Philips versus Reference (**A**) and Emerald versus Reference (**B**). Normalized scratch duration of Philips versus Reference (**C**) and Emerald versus Reference (**D**). Solid blue lines show the line of best fit from mixed-effects linear regression models, and dashed black lines show the identity line (y=x). The plotted values are the normalized scratch durations and normalized scratch frequencies. Each point represents observed scratching from 1 participant night. The x-axis and y-axis use a log-transformed scale with tick labels shown on the original scale. Each point is colored according to the study cohort. AD: atopic dermatitis; HV: healthy volunteer; TSO: total sleep opportunity.

The level of agreement in nocturnal scratch measures between each DHT and Reference on a nightly level is shown in Figure 4. The points were largely distributed along the identity line, indicating that the predicted nocturnal scratch frequency and nocturnal scratch duration were similar to the reference. Philips scratch predictions had a higher false-positive rate, resulting in overestimation of both nocturnal scratch frequency and duration as observed in Figures 4A and 4C. Emerald scratch predictions fall more evenly about the identity line, indicating lower bias as observed in Figures 4B and 4D.

The primary hypothesis of this study was that the nocturnal scratch metrics estimated from DHTs would be comparable with nocturnal scratch metrics assessed by human-labeled IR video. However, to our knowledge, the level of human agreement expected between human raters for this task had not been characterized in the literature and thus was unknown to us at the start of the study. Therefore, we used a rigorous human labeling approach where every night of video was first split into motion and nonmotion periods, and then every motion period was independently labeled by at least 2 human raters for scratching. To quantify the level of agreement between methods, the ICC was used as a measure of interrater reliability.

**Figure 5.**
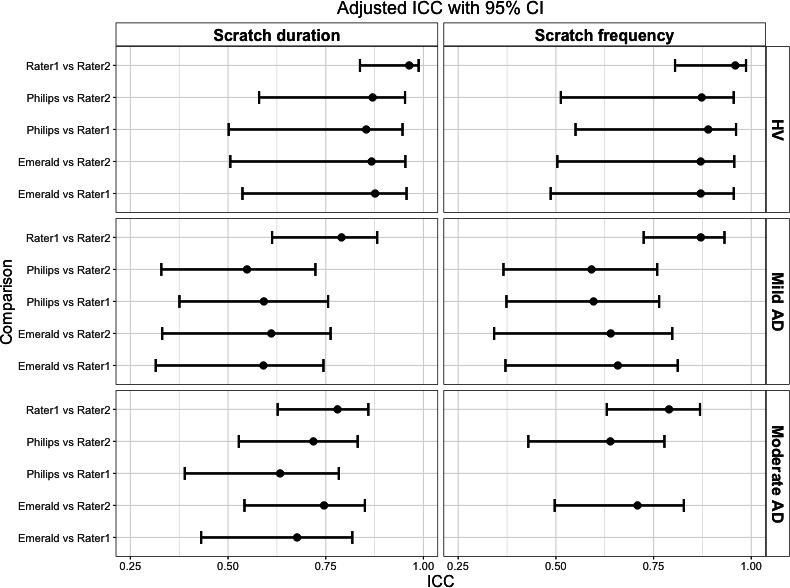
ICC estimates showing agreement between 2 human raters and between each digital health technology and a human rater. Dots show the ICC point estimates and whiskers show the 95% CIs. Note that rater 1 values are missing for normalized scratch frequency in the moderate AD cohort due to nonconvergence of the model. AD: atopic dermatitis; HV: healthy volunteer; ICC: intraclass correlation.

Interrater reliability estimates comparing 2 human raters fell in the “good” to “excellent” range (95% CI lower bound >0.75) for HVs and the “moderate” to “good” range (95% CI lower bound 0.5‐0.9) for patients with AD [28] (Figure 5). In all cohorts, the 95% CIs for DHT versus human rater and human rater versus human rater ICCs overlapped, indicating noninferiority. The noninferior ICC estimates for both Philips and Emerald indicate that DHT estimates of normalized scratch duration and frequency are comparable with human raters.

## Discussion

### Principal Results and Comparison With Prior Work

We conducted an analytical validation study of both a wearable sensor and a touchless sensor, and corresponding algorithms, for the ability to correctly identify nocturnal scratching events in adults with AD, while participants were sleeping at home. We found that the intended sleep window (TSO) can be reliably estimated with both DHTs. The TSO determination for both the Philips DHT and the Emerald DHT was in the range previously reported in a sleep clinic (<30 minutes mean bias) [10]. The sensitivity of scratch classification performance was higher with the Philips DHT, whereas the precision was higher with the Emerald DHT. Overall, the *F*_1_-score was higher with the Emerald DHT (ranging from 0.51 to 0.68) than with the Philips DHT (ranging from 0.47 to 0.56). The Philips algorithm was found to be oversensitive, falsely identifying movements as scratch events during nonscratch participant motion. Participant-level *F*_1_-scores varied widely from 0.13 to 0.85, and this was highly associated with the scratch prevalence—participants having a higher scratch prevalence generally achieved a higher *F*_1_-score for both Emerald and Philips DHT. Other weaker associations identified with the participant-level *F*_1_-scores (accelerometer power, ethnicity, and age) should be interpreted cautiously due to the limited sample size.

While the *F*_1_-scores in this study were lower than those previously reported for these DHTs [12,24], this was expected due to several factors. First, the dataset used was previously unseen by the algorithms tested, whereas previous studies tested an algorithm that was developed on the same dataset. Second, the study execution was made as “real-world” as possible with all data collected in the participants’ homes. Additionally, the comparison with reference to generate *F*_1_-scores was performed on all windows, without first balancing scratch and nonscratch events, which was shown to have a large impact on *F*_1_-score [11]. Finally, we have shown that *F*_1_-score is influenced by scratch prevalence, which makes it difficult to compare results across studies without accounting for differences in the scratch prevalence between study populations.

Importantly, we quantified the interrater agreement for the nightly scratch summary measures and found that the performance of both DHTs and human raters was noninferior to how 2 independent human raters performed when compared with each other. The mean level of human interrater agreement for nocturnal scratch duration and nocturnal scratch frequency was approximately 0.75 for both mild and moderate AD groups. This finding provides a ceiling for the maximal level of agreement expected between DHT and human raters. Our results show that current DHTs are close to, but do not reach, this ceiling of agreement between 2 human raters. Future work should investigate whether improved nocturnal scratching algorithms trained on additional datasets could reach the level of agreement of 2 human raters. Algorithm development should seek to minimize false positives from nonscratch movements.

When characterizing human-labeled scratch measures, we noted that nocturnal scratching was also observed in HVs in this study. While previous studies have generally found little to no scratching among HVs as assessed by video monitoring [9,12,17], recent results from other studies of nocturnal scratching hint that even HVs may scratch during the night [29]. We also found that nocturnal scratch duration and frequency were similar between mild AD and moderate AD cohorts in this study. Previous work has also shown overlapping ranges between mild and moderate AD without finding a significant relationship in the total scratching time as a percentage of the total recording [9]. Converting the metric used in Figure 1 of Ebata et al [9] into normalized scratch duration as we define it would lead to a median estimate of normalized scratching duration of <36 seconds per hour in mild AD and 216 seconds per hour in moderate AD. Therefore, it is possible that our study tended to recruit people who scratch less than a typical patient with moderate AD. Notably, the similarity in mild AD and moderate AD scratch was also mirrored in the self-reported itch NRS at screening, which had a similar range and median value in both cohorts (Table 1). Future work may further elucidate whether there are differences in the characteristics (eg, intensity, distribution by sleep stage, and timing) of scratching between patients with AD of varying severities or between patients with AD as a whole and, otherwise, healthy individuals reporting no pruritus.

### Limitations

This study has a few limitations to be considered. The number of participants analyzed (n=28) was relatively small since it was chosen to be powered primarily for the analysis of ICC estimation in patients with moderate AD. Still, the sample size is comparable with other studies of nocturnal scratching [10-12], and this study was unique in that rather than 1 or 2 nights of collected IR video, participants collected, on average, 8 nights of IR video over the 4-week observation period. The study also had a limited sample size to investigate the performance of the scratch algorithms in subgroups (eg, by age or race). While associations with *F*_1_-scores were performed, additional data are necessary to reach a conclusion about scratch performance in any subgroups. Future work should test these tools in a more diverse population to ensure that performance is acceptable in a representative sample of the targeted population. This study has another limitation tied to the collection of video data in the home. An intentional choice was made to give the study participants full control over the IR video recording, allowing them to turn it on and off at will. This could result in some degree of bias in the determination of the reference TSO window because it is possible that participants recorded video only for part of the true TSO window. Anecdotal evidence from DHT-derived sleep windows suggests that on some nights, the camera may have been turned off before the end of the sleep period, leading to a bias in the Reference TSO duration. To mitigate the impact of this bias, short estimated TSO windows (<3.5 hours) were excluded from the analysis. Overall, the net benefits of performing the study in the home (less participant burden and a natural sleeping environment) outweigh any potential drawbacks in capturing the TSO.

While our intent was to enroll patients with a broad range of disease severity, no patients with AD with severe disease according to vIGA-AD enrolled in our study. This is due, in part, to the requirements for having a stable regimen of systemic treatment for at least 4 weeks prior to study screening and no changes in treatment during the 4-week study, which made it difficult to recruit participants with severe disease. Notably, when classifying the study participants according to SCORAD, there are 9 participants considered severe (total score of >50). Future analytical validation studies may consider modifying the inclusion criteria to recruit participants with a broader range of AD severity.

Results from the Emerald DHT are promising; however, data in this study were collected with strict lifestyle restrictions. Namely, participants were required to sleep without any other people or pets in the bedroom due to the requirements of current algorithms for detecting scratching. Future work should focus on algorithmic solutions to the problem of detecting scratching in a single individual while not sleeping alone to enable scaling this technology for more widespread clinical trial use.

### Conclusions

Our results support the analytical validity of both DHTs tested for continuous measurement of nocturnal scratching in the home environment. The most supportive finding is the noninferior performance of both the Emerald DHT and Philips DHT compared with human labelers. Opportunities remain for improving the performance of the DHTs tested, especially in the precision of wrist-worn accelerometer scratch detection, to reach human-level performance. Nevertheless, ultimately the use of measuring nocturnal scratching as an efficacy end point for clinical trials is dependent not on perfect agreement with the reference but rather on detecting clinically meaningful changes in scratching behavior in patients. Therefore, current tools may be fit-for-purpose to generate clinical trial end points depending on their intended use and the magnitude of the treatment effect on specific nocturnal scratch end points. Practitioners who would like to use these tools may consider collecting additional data on diverse samples to better understand scratching behavior in the context of natural history studies or interventional studies where treatment effects on scratching behavior can be assessed.

## Supplementary material

10.2196/72216 Multimedia Appendix 1 Participant-level within-night scratch classification performance metrics.

10.2196/72216 Multimedia Appendix 2 Sensitivity, precision, *F*_1_-score, and balanced accuracy to detect scratching events on 1-second windows compared to the Reference.

10.2196/72216 Multimedia Appendix 3 Association of scratch detection performance (*F*_1_) with participant-level characteristics.

## Abbreviations

AD: atopic dermatitis
DHT: digital health technology
HV: healthy volunteer
ICC: intraclass correlation coefficient
IR: infrared
NRS: numeric rating scale
PRO: patient-reported outcome
RADA: Rapid Actigraphy Data Analyzer
RF: radio frequency
SCORAD: SCORing Atopic Dermatitis
TSO: total sleep opportunity
vIGA-AD: Validated Investigator Global Assessment scale for Atopic Dermatitis

## References

[1] Bieber T (2008). Atopic dermatitis. N Engl J Med.

[2] Patel T, Ishiuji Y, Yosipovitch G (2007). Nocturnal itch: why do we itch at night?. Acta Derm Venereol.

[3] Cesnakova L, Meadows K, Avey S (2023). A patient-centred conceptual model of nocturnal scratch and its impact in atopic dermatitis: a mixed-methods study supporting the development of novel digital measurements. Skin Health Dis.

[4] Charman CR, Venn AJ, Williams HC (2004). The patient-oriented eczema measure: development and initial validation of a new tool for measuring atopic eczema severity from the patients’ perspective. Arch Dermatol.

[5] Topp J, Apfelbacher C, Ständer S, Augustin M, Blome C (2022). Measurement properties of patient-reported outcome measures for pruritus: an updated systematic review. J Invest Dermatol.

[6] Ke Wang W, Cesnakova L, Goldsack JC, Dunn J (2023). Defining the digital measurement of scratching during sleep or nocturnal scratching: review of the literature. J Med Internet Res.

[7] Yang AF, Nguyen M, Li AW (2021). Use of technology for the objective evaluation of scratching behavior: a systematic review. JAAD Int.

[8] Ebata T, Aizawa H, Kamide R (1996). An infrared video camera system to observe nocturnal scratching in atopic dermatitis patients. J Dermatol.

[9] Ebata T, Aizawa H, Kamide R, Niimura M (1999). The characteristics of nocturnal scratching in adults with atopic dermatitis. Br J Dermatol.

[10] Mahadevan N, Christakis Y, Di J (2021). Development of digital measures for nighttime scratch and sleep using wrist-worn wearable devices. NPJ Digit Med.

[11] Ji J, Venderley J, Zhang H (2023). Assessing nocturnal scratch with actigraphy in atopic dermatitis patients. NPJ Digit Med.

[12] Moreau A, Anderer P, Ross M, Cerny A, Almazan TH, Peterson B (2018). Detection of nocturnal scratching movements in patients with atopic dermatitis using accelerometers and recurrent neural networks. IEEE J Biomed Health Inform.

[13] Padmanabha A, Choudhary S, Majidi C, Erickson Z (2023). A multimodal sensing ring for quantification of scratch intensity. Commun Med.

[14] Chun KS, Kang YJ, Lee JY (2021). A skin-conformable wireless sensor to objectively quantify symptoms of pruritus. Sci Adv.

[15] Yang AF, Chun KS, Yu L (2023). Validation of a hand-mounted wearable sensor for scratching movements in adults with atopic dermatitis. J Am Acad Dermatol.

[16] Ebata T, Iwasaki S, Kamide R, Niimura M (2001). Use of a wrist activity monitor for the measurement of nocturnal scratching in patients with atopic dermatitis. Br J Dermatol.

[17] Ikoma A, Ebata T, Chantalat L (2019). Measurement of nocturnal scratching in patients with pruritus using a smartwatch: initial clinical studies with the itch tracker app. Acta Derm Venereol.

[18] Smith MP, Ly K, Thibodeaux Q (2019). Emerging methods to objectively assess pruritus in atopic dermatitis. Dermatol Ther (Heidelb).

[19] Hsu CY, Ahuja A, Yue S, Hristov R, Kabelac Z, Katabi D (2017). Zero-effort in-home sleep and insomnia monitoring using radio signals. Proc ACM Interact Mobile Wearable Ubiquitous Technol.

[20] Zhao M, Yue S, Katabi D, Jaakkola TS, Bianchi MT (2017). Learning sleep stages from radio signals: a conditional adversarial architecture. https://proceedings.mlr.press/v70/zhao17d.html.

[21] Liu Y, Zhang G, Tarolli CG (2022). Monitoring gait at home with radio waves in Parkinson’s disease: a marker of severity, progression, and medication response. Sci Transl Med.

[22] Benjamin K, Waterston K, Russell M, Schofield O, Diffey B, Rees JL (2004). The development of an objective method for measuring scratch in children with atopic dermatitis suitable for clinical use. J Am Acad Dermatol.

[23] Yang AF, Xu S (2021). 455 Correlation between objective measures of sleep and nocturnal scratch in children with atopic dermatitis. J Invest Dermatol.

[24] Ouroutzoglou M, Zhao M, Hellerstein J (2025). Quantifying itch and its impact on sleep using machine learning and radio signals. arXiv.

[25] Kuznetsova A, Brockhoff PB, Christensen RHB (2017). lmerTest Package: Tests in linear mixed effects models. J Stat Soft.

[26] Lüdecke D, Ben-Shachar M, Patil I, Waggoner P, Makowski D (2021). Performance: an R package for assessment, comparison and testing of statistical models. J Open Sour Softw.

[27] McGraw KO, Wong SP (1996). Forming inferences about some intraclass correlation coefficients. Psychol Methods.

[28] Koo TK, Li MY (2016). A guideline of selecting and reporting intraclass correlation coefficients for reliability research. J Chiropr Med.

[29] Alexopoulos C, Kassim A, Christakis Y (2024). 518—Interaction of sleep disturbance and nocturnal scratch in atopic dermatitis patients. Br J Dermatol.

[30] Clinical trial data transparency. Johnson & Johnson Innovative Medicine.

[31] The Yoda Project.

