# Supplementary material for: At-Home Evaluation of Both Wearable and Touchless Digital Health Technologies for Measuring Nocturnal Scratching in Atopic Dermatitis: Analytical Validation Study
Source: J Med Internet Res. 2025 Jul 15;27:e72216. doi: 10.2196/72216 (PMC12282645; doi:10.2196/72216)
Supplement: Multimedia Appendix 2 [file jmir-v27-e72216-s002.docx]

| Supplementary Table 2: Sensitivity, Precision, F1 Score and Balanced Accuracy to Detect Scratching Events on 1-s Windows Compared to the Reference | | |
| --- | --- | --- |
|  | Emerald | Philips |
| HV |  |  |
| N (Participants) |  |  |
|  | 5 | 5 |
| N (Participant-Nights) |  |  |
|  | 46 | 45 |
| Sensitivity |  |  |
|  | 0.48 | 0.53 |
| Precision |  |  |
|  | 0.51 | 0.34 |
| F1 |  |  |
|  | 0.50 | 0.42 |
| Balanced Accuracy |  |  |
|  | 0.74 | 0.76 |
| Mild AD |  |  |
| N (Participants) |  |  |
|  | 9 | 9 |
| N (Participant-Nights) |  |  |
|  | 76 | 73 |
| Sensitivity |  |  |
|  | 0.50 | 0.43 |
| Precision |  |  |
|  | 0.43 | 0.30 |
| F1 |  |  |
|  | 0.46 | 0.36 |
| Balanced Accuracy |  |  |
|  | 0.75 | 0.71 |
| Moderate AD |  |  |
| N (Participants) |  |  |
|  | 14 | 14 |
| N (Participant-Nights) |  |  |
|  | 98 | 74 |
| Sensitivity |  |  |
|  | 0.68 | 0.57 |
| Precision |  |  |
|  | 0.61 | 0.42 |
| F1 |  |  |
|  | 0.64 | 0.48 |
| Balanced Accuracy |  |  |
|  | 0.84 | 0.78 |
|  | | |
